# Prognostic value of interleukin-34 and interleukin-38 in patients with newly diagnosed atrial fibrillation

**DOI:** 10.3389/fcvm.2022.1072164

**Published:** 2023-01-09

**Authors:** Jiaxue Ma, Na Wu, Zhiquan Yuan, Yanxiu Chen, Chengying Li, Weijia Xie, Zhihui Zhang, Yafei Li, Li Zhong

**Affiliations:** ^1^Cardiovascular Disease Center, Third Affiliated Hospital of Chongqing Medical University, Chongqing, China; ^2^Department of Epidemiology, College of Preventive Medicine, Army Medical University, Chongqing, China; ^3^Evidence-based Medicine and Clinical Epidemiology Center, Army Medical University, Chongqing, China; ^4^Department of Cardiology, Center for Circadian Metabolism and Cardiovascular Disease, Southwest Hospital, Army Medical University, Chongqing, China

**Keywords:** interleukins, prognosis, CHA2DS2-VASc score, ABC score, atrial fibrillation

## Abstract

**Background:**

Interleukin (IL)-34 and IL-38 are associated with cardiovascular disease (CVD). However, their involvement in atrial fibrillation (AF) and AF-associated adverse events remains uncertain. Therefore, we aimed to investigate their association with various AF prognostic factors in a cohort study and assessed their predictive value for the prognosis of patients with AF.

**Methods:**

Patients with new-onset non-valvular AF were consecutively enrolled between 2013 and 2015 at the Department of Cardiovascular Medicine of the Southwest Hospital of the Army Medical University (Third Military Medical University) in Chongqing, China. The endpoints included stroke and all-cause mortality. The baseline levels of plasma IL-34, IL-38, NT-proBNP, high-sensitivity cardiac troponin T (hs-cTnT), and GDF-15 were measured and their correlation with AF-related adverse events were analyzed in a Cox proportional-hazards regression model. The *C*-statistic, net reclassification improvement (NRI), and integrated discrimination improvement (IDI) were used to evaluate the performance of the AF prognostic models. Decision curve analysis (DCA) was used to evaluate the clinical net benefit of the original and modified models.

**Results:**

A total of 299 patients with new-onset AF were enrolled. During the median follow-up time of 28 (IQR: 27, 29) months, the higher levels of IL-34 were associated with a lower risk of stroke, and the higher levels of IL-38 were associated with an increased risk of all-cause death (all adjusted *P* < 0.05). In addition, elevated hs-cTnT and NT-proBNP concentrations were associated with a higher risk of stroke and all-cause mortality (all adjusted *P* < 0.05). Furthermore, the CHA_2_DS_2_-VASc score combined with IL-38 and NT-proBNP significantly improved the *C*-statistic, IDI, and NRI (all *P* < 0.01). There was no statistically significant difference (all *P* > 0.05) in the discrimination power between the preference models and the ABC (age, biomarkers, and clinical history) score for the two prognostic outcomes.

**Conclusion:**

Our results suggested that IL-34 and IL-38 were independently associated with stroke and all-cause mortality in patients with AF. Moreover, adding IL-38 and NT-proBNP to the CHA_2_DS_2_-VASc score significantly improved its predictive ability of AF-related all-cause death. Finally, the preference model performed equally well as the ABC score in predicting AF prognosis.

## 1. Introduction

Atrial fibrillation (AF) is the most common cardiac arrhythmia which has become a major public health concern worldwide, and its prevalence is projected to increase further in the coming years (1). The onset of AF and its development is usually accompanied by many adverse events, which can lead to disability and mortality (2). Therefore, prediction and risk stratification of prognosis is particularly essential for better monitoring and management of AF. Risk stratification patterns recommended by current AF guidelines are mainly based on clinical risk factors, such as the CHA_2_DS_2_-VASc score (3). However, the identification power of CHA_2_DS_2_-VASc score is still limited in estimating the “truly high-risk” subjects, with a *C*-statistic of only 0.6 (4). Multiple studies have found biomarkers carrying rich prognostic information in AF (5–7). Hijazi et al. developed a new biomarker-based model in 2016, the ABC (age, biomarkers, and clinical history) score, which yielded a higher *C*-statistic than the CHA_2_DS_2_-VASc score (8).

Circulating interleukins (ILs) are a class of cytokines involved in information transmission, activation, and inflammatory responses, which have been intensively investigated in cardiovascular disease (CVD) over the last few years (9–11). Among them, IL-34 has been shown associated with CVD risk (12, 13), and IL-38 may be a key regulator of CVD (14). At present, relatively little is known about the role of IL-34 and IL-38 in AF-associated adverse events. In addition, the question remains to be answered whether IL-34 and IL-38 can improve the predictive power of the existing risk stratification scheme (CHA_2_DS_2_-VASc score) for AF-related prognosis.

Therefore, we aimed to explore the associations between ILs and stroke and all-cause death and to evaluate their predictive value in AF outcomes. Furthermore, we compared the prognostic predictive power of CHA_2_DS_2_-VASc score combined with IL-34 or IL-38 to that of the ABC score, to find new prognosis-associated biomarkers of AF and improve prognostic assessment of AF patients.

## 2. Materials and methods

### 2.1. Study design and populations

We conducted a cohort study. Patients aged greater than or equal to 18 years with new-onset AF were consecutively enrolled between December 2013 and August 2015 at the Department of Cardiovascular Medicine of the Southwest Hospital of the Army Medical University (Third Military Medical University) in Chongqing, China. The diagnostic criteria of AF were based on the definition by the 2020 ESC Guidelines (15). Patients were excluded if they met one of the following criteria: moderate-to-severe mitral stenosis, artificial valve replacement, malignant tumors, acute and chronic inflammatory diseases, connective tissue diseases, and/or infections. The study complied with the principles of the Declaration of Helsinki and was approved by the Ethics Committee of Southwest Hospital of the Army Medical University. Informed consents have been obtained from all subjects.

### 2.2. The CHA_2_DS_2_-VASc and ABC scores

The CHA_2_DS_2_-VASc score was calculated using clinical information [heart failure, hypertension, diabetes, vascular disease, aged 65–74 years old, female accounted for one point. Age ≥ 75 years old, and stroke history/transient ischemic attack (TIA)/thromboembolism history accounted for two points] (3). The ABC-stroke score was calculated according to the study of Hijazi et al. [age, the levels of troponin (Tn) T/I, NT-proBNP, prior stroke/systemic embolism (SE), and 1-year stroke risk incidence rate of individual] (8). Also, the ABC-death score was calculated according to the study of Hijazi et al. (16) (age, the levels of Tn T/I, NT-proBNP, GDF-15, the history of heart failure, and 1-year mortality risk incidence rate of individual).

### 2.3. Measurement of the levels of biomarkers and interleukins

Fasting blood samples (5 ml) were drawn from all participants (within 48 h after admission and before any treatments). Plasma fractions were obtained immediately by centrifugation at 2,000 rpm/min for 15 min at 4°C and stored in a −80°C freezer until use.

NT-proBNP and high-sensitivity cardiac troponin T (hs-cTnT) levels were measured using an electro-chemiluminescence immunoassay (Cobas e601, Rocha Diagnostics, Manheim, Germany). The detection range of NT-proBNP and hs-cTnT were 5–35,000 pg/ml and 3–10,000 ng/L, respectively. GDF-15 levels were assessed by enzyme-linked immunosorbent assay (ELISA) kit (RayBiotech, Norcross, GA, USA), and the analytical range was 2–800 pg/ml, the inter-assay and intra-assay coefficient of variation were less than 10 and 12%, respectively. Measurements of IL-34 and IL-38 were performed with Bio-plex Pro™ plex xMAP array technology (Bio-Rad Corporation, Hercules, CA, USA) based on Luminex 200 system (Luminex Corporation, Austin, TX, USA), and the concentrations were calculated by fluorescence intensities from the corresponding standard curves, based on Bio-Plex Manager™ 6.1 (Bio-Rad) software. Each sample was tested once. The standard and reference products were used for quality control, all of which showed coefficients of variation of less than 12.1%.

### 2.4. Endpoint events and follow-up

The primary endpoint was stroke (ischemic and hemorrhagic) and the secondary endpoint was all-cause death. The follow-up time for each individual was recorded from the date of entry to the date of death, or the end of the trial. The patient’s physical conditions or causes of death were verified annually through electronic medical records, reports of close relatives, and national death registration system.

### 2.5. Statistical analyses

The Shapiro–Wilk test was used to test if continuous variables follow a normal distribution. Continuous variables were presented by mean ± SD or median with interquartile ranges (*IQRs*). Categorical variables were presented as numbers and percentages. The optimal cut-off values of biomarkers were determined by X-tile software (Yale University, New Haven, CT, USA) (17). To avoid multiple cut-off values in this study, we considered stroke and all-cause death as a composite endpoint.

To investigate the associations between biomarkers and AF prognosis, biomarkers were initially examined by univariate Cox proportional-hazard analysis. Then, the variables with *P* < 0.10 in univariate Cox proportional-hazard analysis were incorporated into the multivariate Cox proportional-hazard analysis by the forward and likelihood ratio (LR) method. In addition, we used the same method (forward and LR) for sensitivity analysis to verify the robustness of the above results. Sensitivity analysis was performed with adjustment for the AF-related covariates: warfarin, statins, and angiotensin receptor inhibitors (ARB) and the components of the CHA_2_DS_2_-VASc score. The variance inflation factor (VIF) was used to examine the multicollinearity of the variables.

The value of *C*-index (Harrell’s C) (18), integrated discrimination improvement (IDI), and net reclassification improvement (NRI) were calculated to evaluate the predictive power of the models in discrimination and reclassification (19). Decision curve analysis (DCA) as described by Vickers et al. (20) was used to quantify the clinical net benefit of each model and to visually compare models.

A two-sided *p*-value < 0.05 was considered statistically significant. All analyses were performed with SPSS statistical software version 25.0 (SPSS Inc., Chicago, IL, USA.) and R software version 4.1.2 (R Project for Statistical Computing, Vienna, Austria).

## 3. Results

### 3.1. Baseline characteristics of study participants

A total of 299 participants underwent this trial (Supplementary Figure 1). Among them, 132 (44.15%) were female, the median age was 66 years (*IQR*: 58, 73). 25 (8.36%) patients were lost to follow-up. The demographic and clinical characteristics of the participants at baseline were presented in Table 1. During the median follow-up time of 28 (*IQR*: 27, 29) months, we identified 26 strokes (4.12 per 100 person-years) and 30 deaths (4.48 per 100 person-years).

**TABLE 1:** Demographic and clinical data of atrial fibrillation patients at baseline.

Characteristics	Value (*n* = 299)
**Age (years)**	66 (58, 73)
<65	130 (43.48%)
65–74	101 (33.78%)
≥75	68 (22.74%)
**Gender**	
Male	167 (55.85%)
Female	132 (44.15%)
**BMI (kg/m^2^)**	23.9 (21.8, 26.2)
<24.9	185 (61.87%)
25.0−29.9	92 (30.77%)
≥30.0	22 (7.36%)
**Education**	
Junior middle school or below	244 (81.61%)
High school or above	55 (18.39%)
**The income per head (10,000 yuan/year)**	
<2.5	148 (49.50%)
≥2.5	151 (50.50%)
**AF types**	
Paroxysmal AF	100 (33.44%)
Chronic AF	199 (66.56%)
Smoking	100 (33.44%)
Alcohol consumption	94 (31.44%)
**History of comorbidities**	
Hypertension	153 (51.17%)
Diabetes mellitus	60 (20.07%)
CAD	124 (41.47%)
Cardiomyopathy	35 (11.71%)
HF	99 (33.11%)
TIA or previous stroke	41 (13.71%)
Vascular disease	18 (6.02%)
**Concomitant treatment**	
Antiarrhythmic therapy	146 (48.83%)
ACEI	107 (35.79%)
ARB	29 (9.70%)
Beta-blockers	119 (39.80%)
Warfarin	96 (32.11%)
Statins	144 (48.16%)
Ablation	71 (23.7%)
LAAC	13 (4.3%)
**Echocardiography parameters**	
LVEF (%)	59 (50, 64)
LA diameter (mm)	44.00 (40.00, 51.00)
**Biomarkers**	
NT-proBNP (pg/ml)	1,083.90 (447.30, 2,154.00)
≤3,580.17	255 (85.28%)
>3,580.17	44 (14.72%)
hs-cTnT (ng/ml)	11.00 (7.00, 25.00)
≤11.00	155 (51.84%)
>11.00	144 (48.16%)
GDF-15 (pg/ml)	1,028.52 (742.12, 1,485.00)
≤1,813.62	250 (83.61%)
>1,813.62	49 (16.39%)
**Interleukin**	
IL-34 (pg/ml)	537.03 (13.93, 791.94)
≤138.47	90 (30.10%)
>138.47	209 (69.90%)
IL-38 (pg/ml)	17.75 (6.32, 33.07)
≤58.25	268 (89.63%)
>58.25	31 (10.37%)

Continuous variables were presented as median with interquartile ranges (IQRs). Categorical variables were presented as numbers and percentages. BMI, body mass index; AF, atrial fibrillation; CAD, coronary artery disease; HF, heart failure; TIA, transient ischemic attack; ACEI, angiotensin-converting enzyme inhibitors; ARB, angiotensin-renin blockers; LAAC, percutaneous left atrial appendage closure; LVEF, left ventricular ejection fraction; LA, left atrium; NT-proBNP, N-terminal fragment B-type natriuretic peptide; hs-cTnT, high-sensitivity cardiac troponin T; GDF, growth differentiation factor; IL, interleukin.

### 3.2. Association of biomarkers with AF prognosis

The optimal cut-off values of biomarkers were determined by the X-tile software based on follow-up time, composite endpoint event (stroke and all-cause death). Optimal cut-off values of IL-34, IL-38, NT-proBNP, hs-cTnT, and GDF-15 were 138.47 pg/ml, 58.25 pg/ml, 3,580.17 pg/ml, 11.00 ng/ml, and 1,813.62 pg/ml, respectively (Supplementary Table 1).

We incorporated variables with *P* < 0.10 in univariate Cox regression (Supplementary Table 2) into the multivariate Cox regression model. In addition, the treatments (warfarin, statins, and ARB) and the components of the CHA_2_DS_2_-VASc score were further adjusted in the sensitive analysis and no differences were found. The collinearity was ignored in this study as the VIFs of variables were less than 2.0 in all models. We found that IL-34 [hazard ratio (HR): 0.36, 95% confidence interval (CI): 0.17–0.78, *P* = 0.010] and hs-cTnT (HR: 3.09, 95% CI: 1.33−7.19, *P* = 0.009) were independently correlated with the risk of stroke. For all-cause mortality, patients with higher levels of IL-38 (HR: 3.11, 95% CI 1.16−8.29, *P* = 0.024) and NT-proBNP (HR: 2.77, 95% CI 1.13−6.78, *P* = 0.025) had an increased risk of all-cause death (Table 2). Although higher GDF-15 level was related to a higher risk of all-cause death in unadjusted analysis, we found that it was not an independent risk factor for AF-related prognosis in the adjusted model.

**TABLE 2:** Associations between biomarkers concentrations and events during follow-up in atrial fibrillation (AF) patients.

Endpoint	Biomarkers	Model*		
		Beta coefficients	Adjusted HR (95% CI)	*P*-value
Stroke	IL-34 (>138.47 pg/ml)	−1.019	0.36 (0.17−0.78)	**0.010**
	hs-cTnT (>11.00 ng/ml)	1.130	3.09 (1.33−7.19)	**0.009**
All-cause mortality	IL-38 (>58.25 pg/ml)	1.133	3.11 (1.16−8.29)	**0.024**
	NT-proBNP (>3,580.17 pg/ml)	1.020	2.77 (1.13−6.78)	**0.025**

Bold indicates *P* < 0.05. HR, hazard ratio; CI, confidence interval; ILs, interleukins; NT-proBNP, N-terminal fragment B-type natriuretic peptide; hs-cTnT, high-sensitivity cardiac troponin T. *Cox proportional-hazard model for stroke adjusted for age, diabetes, stroke/TIA/thromboembolism, left atrium (LA), and use of angiotensin-converting enzyme inhibitors (ACEI); for all-cause mortality adjusted for age, body mass index (BMI), types of AF, heart failure, left ventricular ejection fraction (LVEF), LA, hs-cTnT, and growth differentiation factor (GDF)-15.

### 3.3. Incremental prognostic value of CHA_2_DS_2_-VASc combined with biomarkers for AF

**FIGURE 1:**
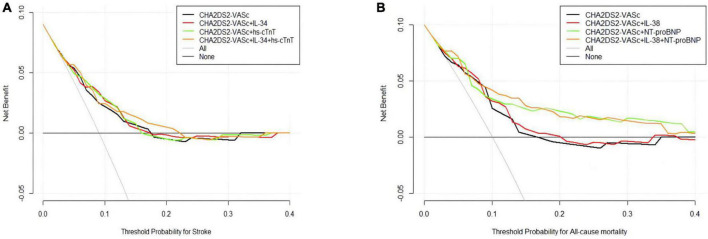
Decision curve analysis (DCA) of the original and modified CHA_2_DS_2_-VASc score. **(A)** Stroke model added hs-cTnT and/or IL-34; **(B)** all-cause mortality model added IL-38 and/or NT-proBNP. The black lines (original scores) and other lines (modified scores) represent the net benefit of each model graphically. The horizontal axis represents the threshold probability of the target adverse event risk. The longitudinal axis shows the net benefit. ILs, interleukins; NT-proBNP, N-terminal fragment B-type natriuretic peptide; hs-cTnT, high-sensitivity cardiac troponin.

We assessed the predictive capabilities of the CHA_2_DS_2_-VASc model combined with biomarkers (Table 3). For all-cause death risk stratification, the CHA_2_DS_2_-VASc integrated IL-38 and NT-proBNP, achieved a *C*-statistic of 0.80 (95% CI 0.73−0.87), which had a significant improvement over the original model (*C*-statistic: 0.70, 95% CI 0.63−0.77, *P* = 0.005), and the reclassification ability was significantly better (IDI: 3.7%, 95% CI: 1.1−13.3%, *P* < 0.001; NRI: 77.6%, 95% CI: 21.9−82.7%, *P* < 0.001). On the other hand, the CHA_2_DS_2_-VASc score combined with IL-34 and hs-cTnT for stroke risk stratification achieved highest *C*-statistic of 0.75 (95% CI 0.66−0.83), which did not show a significant statistical difference from other scores. The DCA visualized the clinical net benefit of the original and modified CHA_2_DS_2_-VASc score (Figure 1). For predicting stroke, the clinical net benefit of modified CHA_2_DS_2_-VASc score adding IL-34 and hs-cTnT was slightly better than other scores (Figure 1A). For predicting all-cause death, adding IL-38 and NT-proBNP to CHA_2_DS_2_-VASc had the best clinical net benefit when the threshold probability was between 9 and 35% (Figure 1B).

**TABLE 3:** Discrimination and reclassification for stroke, all-cause mortality, or cardiovascular death.

	Discrimination			Reclassification			
	C-statistic (95% CI)	Improvement in C-statistic (95% CI)	*P*-value	IDI (%) (95% CI)	*P*-value	NRI (%) (95% CI)	*P*-value
**Stroke**							
CHA_2_DS_2_-VASc	0.71 (0.63, 0.79)						
CHA_2_DS_2_-VASc + IL-34	0.72 (0.64, 0.81)	0.01^#^ (−0.04, 0.05)	0.533	0.2^#^ (−0.2, 1.7)	0.498	20.3^#^ (−28.7, 68.2)	0.557
CHA_2_DS_2_-VASc + hs-cTnT	0.73 (0.66, 0.81)	0.02^#^ (−0.03, 0.07)	0.303	0.3^#^ (0.0, 1.8)	0.070	52.6^#^ (0.0, 57.9)	**0.010**
CHA_2_DS_2_-VASc + IL-34 + hs-cTnT	0.75 (0.66, 0.83)	0.04^#^ (−0.04, 0.09)	0.253	0.8^#^ (0.0, 4.2)	0.060	18.9^#^ (−23.5, 69.3)	0.219
ABC-stroke	0.73 (0.63, 0.82)	−0.02^a^ (−0.10, 0.08)	0.663				
**All-cause mortality**							
CHA_2_DS_2_-VASc	0.70 (0.63, 0.77)						
CHA_2_DS_2_-VASc + IL-38	0.72 (0.65, 0.80)	0.02^#^ (−0.03, 0.07)	0.376	1.2 (−0.1, 6.4)	0.080	40.2^#^ (−20.6, 79.4)	0.090
CHA_2_DS_2_-VASc + NT-proBNP	0.78 (0.71, 0.85)	0.08^#^ (0.02, 0.14)	**0.014**	3.4^#^ (0.0, 12.2)	0.050	35.8^#^ (−4.2, 72.9)	0.090
CHA_2_DS_2_-VASc + IL-38 + NT-proBNP	0.80 (0.73, 0.87)	0.10^#^ (0.03, 0.17)	**0.005**	3.7^#^ (1.1, 13.3)	**<0.001**	77.6^#^ (21.9, 82.7)	**<0.001**
ABC-death	0.83^b^ (0.76, 0.89)	0.02^b^ (−0.04, 0.10)	0.472				

Bold indicates *P* < 0.05, the improvement of the C-statistic is statistically significant.

IDI, integrated discrimination improvement; NRI, net reclassification improvement; CI, confidence interval; ILs, interleukins; NT-proBNP, N-terminal fragment B-type natriuretic peptide; hs-cTnT, high-sensitivity cardiac troponin T.

^#^Comparison with CHA_2_DS_2_-VASc; ^a^Comparison with CHA_2_DS_2_-VASc + IL-34 + hs-cTnT; ^b^Comparison with CHA_2_DS_2_-VASc + IL-38 + NT-proBNP.

## 4. Discussion

In this study, we first explored the association of IL-34 and IL-38 with stroke and all-cause mortality in patients with new-onset AF and found that low levels of IL-34 were independent predictors of stroke, while high concentrations of IL-38 were correlated with all-cause death, which goes beyond earlier findings. In addition, we evaluated whether the addition of IL-34 and IL-38 could improve the predictive performance of the existing prognostic prediction model (CHA_2_DS_2_-VASc score). We showed significant improvement for the all-cause death risk prediction when IL-38 and NT-proBNP were added to the original model (CHA_2_DS_2_-VASc score) Finally, the preferred models in two adverse events predictive models performed equally well as the ABC score.

The pathophysiology of AF is complex and yet to be explored. Several studies have depicted correlations between AF and different inflammatory markers and mediators (21, 22). Tn elevation was initially seen as a sensitive indicator of myocardial damage and infarction. Recent data has shown that cardiac troponin I (cTnI) and T (cTnT) provide important prognostic information in anticoagulated patients with AF for predicting all-cause mortality, cardiac death, stroke or SE (23), which was consistent with our findings that hs-cTnT was independently related to stroke. NT-proBNP, which is degraded by pro-BNP, has been used as a biomarker for heart failure and renal insufficiency. In recent years, its prognostic values have also been applied to AF. The concentrations of NT-proBNP increased during states of hemodynamic stress such as in heart failure, acute coronary syndrome, and arrhythmias including AF (22). In a sub-study of RE-LY, increased concentrations of NT-proBNP were generally associated with the risk of stroke and mortality (24). The same conclusion was obtained in our study. GDF-15 is a member of the transforming growth factor-β (TGF-β) superfamily, which plays an important role as an inflammatory marker in tumor pathogenesis, and ischemic, and metabolic diseases. In addition, it has been widely studied in the field of CVDs. It is not expressed in healthy adult myocardium but significantly expressed in cardiomyocytes, adipocytes, macrophages, endothelial cells, and vascular smooth muscle cells after myocardial lesion (25). In our study, GDF-15 was a risk predictor for mortality on top of clinical characteristics. However, NT-proBNP, hs-TnT, and GDF-15 partly reflected the same processes, which was myocardial dysfunction and cardiovascular comorbidity (7). This might explain why in the adjusted model GDF-15 did not appear to be an independent risk indicator for all-cause death when other biomarkers and clinical characteristics have been taken into account.

Interleukin (IL)-34 as a novel ligand of CSF-1R was defined in 2008 (26), and its biology and function have been broadly studied. In physiologic cases, they perform critical roles in the development of microglia and Langerhans cells (LCs). They also play crucial roles in pathological conditions, such as inflammatory diseases. Over the past few years, its role in CVD has also been investigated and found to be significantly increased in patients with coronary heart disease (CHD), and its increase is positively correlated with the level of high-sensitivity C-reactive protein (hs-CRP), the results of which give evidence for IL-34 as a pro-inflammatory cytokine (27). Moreover, studies have shown that IL-34 may be an important predictor of CVD, heart failure hospitalization, and all-cause mortality in patients with chronic heart failure (CHF) (28). Concurrently, we found that it can be used as an independent predictor for stroke events in AF patients, which may provide a new insight connecting AF to stroke outcomes beyond other biomarkers. However, it was noteworthy that AF patients with lower IL-34 levels have a higher risk of stroke, which seemed counterintuitive to its pro-inflammatory role. On the one hand, the levels of IL-34 were measured only once at the baseline, failing to evaluate the impact of dynamic changes of them on the prognosis. Therefore, the effects of IL-34 level fluctuation on the outcomes of AF need to be further explored in the future. On the other hand, some previous studies also reported that elevated IL-34 may act as a protective factor. Esaki et al. (29) identified decreased expression of IL-34 in atopic dermatitis (AD) compared to non-lesional AD and normal epidermis. The study by Mizuno et al. (30) demonstrated that *in vitro*, microglia treated with IL-34 attenuated the neurotoxic effects of oligomeric amyloid-β (oAβ), which mediates synaptic dysfunction and neuronal damage in Alzheimer’s disease. Moreover, intracerebroventricular administration of IL-34 improves deficits in associative learning. In addition, IL-34 also has a protective role in some cancer, such as non-small cell lung cancer (31), colorectal cancer (32), breast cancer (33, 34), and lung cancer (32), hematologic malignancies (32), and head and neck cancer (33, 34). Therefore, the issue of whether IL-34 is beneficial or harmful in stroke-attacked AF patients merits further study.

Interleukin (IL)-38 (IL-1F10 or IL-1HY2) belongs to IL-36, one of the family members of IL-1. Its primary biological function is to block the activation of the IL-36R signaling pathway and influence the proinflammation function of IL-36, which is similar to IL-36Ra (35). IL-38 is expressed in the thymus, heart, placenta, and fetal liver in healthy conditions (36), whereas in disease it is predominantly expressed in settings in IL-1-driven inflammatory response, such as CVD (37). Previous study indicated that the expression of the IL-38 gene was increased in peripheral blood mononuclear cells (PBMCs) of patients with ST-segment elevation myocardial infarction (STEMI) and had been positively correlated with CRP, cTnI, and NT-proBNP (37).

The CHA_2_DS_2_-VASc score, the main method recommended by current guidelines for predicting stroke outcomes in AF, is derived from the CHADS_2_ score [congestive heart failure, hypertension, age ≥ 75 years, diabetes mellitus, previous stroke (double weight)], and it is better at identifying “truly low risk” (CHA_2_DS_2_-VASc score = 0–1) than the CHADS_2_ score (38). However, as research continues to unfold, it has been found that there was still room for improvement in terms of predicting the “truly high-risk” patients since the *C*-statistic of the CHA_2_DS_2_-VASc score was only 0.60 (95% CI 0.57−0.64) (3). The discovery that biomarkers provide rich information for AF prognosis has led to new strategies to improve prognosis stratification. Hijazi et al. developed an ABC score based on age, biomarkers and clinical history, which had a *C*-statistic of 0.65 (95% CI: 0.61−0.69) and were well-validated both internally and externally (8). In our findings, the *C*-statistic of stroke prediction model integrated IL-34 and/or hs-cTnT were all superior to the original model, and NRI of the CHA_2_DS_2_-VASc score combined with hs-cTnT had a significantly improved. Furthermore, when NT-proBNP was added to the CHA_2_DS_2_-VASc score for predicting all-cause mortality, the *C*-statistic was significantly improved in the new model. In addition, when IL-38 and NT-proBNP were simultaneously added to the CHA_2_DS_2_-VASc score, the *C*-statistic increased, and NRI, and IDI both showed significant improvements of the new model regarding prognostic prediction value. Finally, we also compared the new model with the largest C-statistic to the ABC score and found that they have comparable discrimination capacity, which indicated that our improvements to the current clinical model were acceptable.

## 5. Limitations

There were some limitations of our study. First, we selected participants from a single center which lacks external validity. Therefore, large-scale prospective validation is required in multicenter studies. Second, our follow-up time was relatively short, and the observation of end-point events was insufficient, so it is still necessary to extend the follow-up time to obtain more accurate results. Finally, further studies are needed on the exact biological mechanism of IL-34 and IL-38 in the pathology of AF.

## 6. Conclusion

In conclusion, serum IL-34 and IL-38 may serve as biomarkers of prognostic evaluation for stroke and all-cause mortality in patients with AF. The addition of IL-38 improved the predictive power of the existing model (CHA_2_DS_2_-VASc score) and increases the net clinical benefit. The new model showed comparable prognostic value to the ABC score. Our findings may help with clinical management and prognostic prediction of patients with AF.

## Data availability statement

The original contributions presented in this study are included in the article/Supplementary material, further inquiries can be directed to the corresponding authors.

## Ethics statement

The studies involving human participants were reviewed and approved by the Army Medical University. The patients/participants provided their written informed consent to participate in this study.

## Author contributions

LZ, ZZ, and YL designed this study. JM, NW, and ZY drafted the article and analyzed the data. YC and CL were in charge of data collection. WX did the critical revision of article. All authors have read and approved the final manuscript.
