# Supplementary material for: Prognostic value of interleukin-34 and interleukin-38 in patients with newly diagnosed atrial fibrillation
Source: Front Cardiovasc Med. 2023 Jan 9;9:1072164. doi: 10.3389/fcvm.2022.1072164 (PMC9868840; doi:10.3389/fcvm.2022.1072164)

**Supplementary Table 1** The optimal cut-off values of biomarkers

| Biomarker | Cut-off |
| --- | --- |
| IL-34 | 138.47 pg/ml |
| IL-38 | 58.25 pg/ml |
| NT-proBNP | 3580.17 pg/ml |
| hs-cTnT | 11.00 ng/ml |
| GDF-15 | 1813.62 pg/ml |

Abbreviations: IL, interleukin; NT-proBNP, N-terminal fragment B-type natriuretic peptide; hs-cTnT, high-sensitivity cardiac troponin T; GDF, Growth differentiation factor.

**Supplementary Table 2** Univariate Cox regression analysis of stroke and all-cause mortality during follow-up in atrial fibrillation patients

| Characteristics | Stroke | | |  | All-cause mortality | | |
| --- | --- | --- | --- | --- | --- | --- | --- |
|  | Beta  coefficients | HR  (95%CI) | *P*  value |  | Beta  coefficients | HR  (95%CI) | *P*  value |
| Age (years)  <65  65-74  ≥75 | Reference  0.878  0.546 | ─  2.41 (0.95-6.12)  1.73 (0.75-4.00) | ─  **0.065**  0.201 |  | Reference  0.886  1.488 | ─  2.43 (0.81-7.24)  4.43 (2.12-9.24) | ─  0.112  **<0.001** |
| Gender  Male  Female | Reference  -0.102 | ─  0.90 (0.42- 1.95) | ─  0.794 |  | Reference  0.274 | ─  1.32 (0.64-2.69) | ─  0.453 |
| BMI (kg/m^2^)  <24.9  25.0-29.9  ≥30.0  Education | Reference  -0.602  -0.642 | ─  0.55 (0.22-1.37)  0.53 (0.07-3.94) | ─  0.196  0.532 |  | Reference  -0.681  1.306 | ─  0.51 (0.19-1.36)  3.69 (1.44-9.45) | ─  0.176  **0.006** |
| Junior middle school or below | Reference | ─ | ─ |  | Reference | ─ | ─ |
| High school or above | -0.001 | 1.00 (0.38-2.64) | 0.999 |  | -0.387 | 0.68 (0.24-1.95) | 0.471 |
| The income per head (10000yuan/year) |  |  |  |  |  |  |  |
| <2.5 | Reference | ─ | ─ |  | Reference | ─ | ─ |
| ≥2.5 | 0.393 | 1.48 (0.69-3.19) | 0.316 |  | 0.409 | 1.51 (0.73-3.13) | 0.272 |
| AF types |  |  |  |  |  |  |  |
| Paroxysmal AF | Reference | ─ | ─ |  | Reference | ─ | ─ |
| Chronic AF | 0.251 | 1.29 (0.56-2.94) | 0.551 |  | 0.964 | 2.62 (1.00-6.85) | **0.049** |
| Smoking | -0.039 | 0.96 (0.43-2.14) | 0.925 |  | -0.042 | 0.96 (0.45-2.05) | 0.914 |
| Alcohol consumption | -0.505 | 0.60 (0.24-1.50) | 0.276 |  | -0.071 | 0.93 (0.43-2.03) | 0.858 |
| History of comorbidities |  |  |  |  |  |  |  |
| Hypertension | 0.354 | 1.43 (0.66-3.07) | 0.366 |  | 0.379 | 1.46 (0.70-3.03) | 0.309 |
| Diabetes mellitus | 1.100 | 3.01 (1.39-6.48) | **0.005** |  | 0.410 | 1.51 (0.67-3.38) | 0.321 |
| CAD | 0.469 | 1.60 (0.75-3.40) | 0.224 |  | 0.494 | 1.64 (0.80-3.36) | 0.177 |
| Cardiomyopathy | -0.055 | 0.95 (0.29-3.14) | 0.928 |  | 0.413 | 1.51 (0.58-3.95) | 0.399 |
| HF | 0.116 | 1.12 (0.50-2.50) | 0.777 |  | 1.985 | 7.28 (3.12-16.96) | **<0.001** |
| TIA or previous stroke | 1.287 | 3.62 (1.27-8.06) | **0.002** |  | -1.505 | 0.22 (0.03-1.63) | 0.139 |
| Vascular disease | 0.745 | 2.11 (0.64-7.00) | 0.224 |  | 0.121 | 1.13 (0.27-4.74) | 0.869 |
| Concomitant treatment  Antiarrhythmic therapy | 0.397 | 1.49 (0.69-3.20) | 0.311 |  | -0.383 | 0.68 (0.33-1.42) | 0.304 |
| ACEI | -0.923 | 0.40 (0.15-1.05) | **0.063** |  | 0.177 | 1.19 (0.58-2.48) | 0.636 |
| ARB | 0.420 | 1.52 (0.53-4.40) | 0.438 |  | -0.030 | 0.97 (0.29-3.20) | 0.961 |
| Beta-blockers | 0.483 | 1.62 (0.76-3.45) | 0.210 |  | 0.134 | 1.14 (0.56-2.36) | 0.715 |
| Warfarin | 0.042 | 1.04 (0.47-2.32) | 0.918 |  | 0.205 | 1.23 (0.58-2.58) | 0.588 |
| Statins | 0.026 | 1.03 (0.48-2.18) | 0.947 |  | 0.222 | 1.25 (0.61-2.56) | 0.543 |
| Ablation  LAACs | -3.059  0.619 | 0.05 (0.00-315.09)  1.86 (0.44-7.83) | 0.496  0.399 |  | -3.057  -3.069 | 0.05 (0.00-240.98)  0.05 (0.00-104.93) | 0.483  0.436 |
| Echocardiography parameters |  |  |  |  |  |  |  |
| LVEF (%) | -0.006 | 0.99 (0.96-1.03) | 0.730 |  | -0.062 | 0.94 (0.92-0.97) | **<0.001** |
| LA diameter (mm) | 0.040 | 1.04 (1.00-1.09) | **0.065** |  | 0.049 | 1.05 (1.01-1.09) | **0.010** |
| Biomarkers |  |  |  |  |  |  |  |
| IL-34 (pg/ml) |  |  |  |  |  |  |  |
| ≤138.47 | Reference | ─ | ─ |  | Reference | ─ | ─ |
| >138.47 | -0.931 | 0.39 (0.78-0.84) | **0.016** |  | -0.074 | 0.93 (0.42-2.03) | 0.853 |
| IL-38 (pg/ml) |  |  |  |  |  |  |  |
| ≤58.25 | Reference | ─ | ─ |  | Reference | ─ | ─ |
| >58.25  NT-proBNP (pg/ml) | 0.178 | 1.195 (0.36-3.97) | 0.771 |  | 0.854 | 2.33 (0.95-5.70) | **0.064** |
| ≤3580.17 | Reference | ─ | ─ |  | Reference | ─ | ─ |
| >3580.17 | 0.715 | 2.05 (0.82-5.07) | 0.123 |  | 1.793 | 6.01 (2.93-12.33) | **<0.001** |
| hs-cTnT (ng/ml) |  |  |  |  |  |  |  |
| ≤11.00 | Reference | ─ | ─ |  | Reference | ─ | ─ |
| >11.00 | 0.874 | 2.40 (1.08-5.34) | **0.032** |  | 1.725 | 5.61 (2.15-14.66) | **<0.001** |
| GDF-15 (pg/ml) |  |  |  |  |  |  |  |
| ≤1813.62 | Reference | ─ | ─ |  | Reference | ─ | ─ |
| >1813.62 | 0.582 | 0.79 (0.72-4.41) | 0.209 |  | 1.636 | 5.14 (2.50-10.53) | **<0.001** |

Bold indicates *P*<0.10;

Abbreviations: BMI, body mass index; AF, atrial fibrillation; CAD, coronary artery disease; HF, heart failure; TIA, transient ischemic attack; ACEI, angiotensin-converting enzyme inhibitors; ARB, angiotensin-renin blockers; LAAC, percutaneous left atrial appendage closure; LVEF, left ventricular ejection fraction; LA, left atrium; NT-proBNP, N-terminal fragment B-type natriuretic peptide; hs-cTnT, high-sensitivity cardiac troponin T; GDF, Growth differentiation factor; IL, interleukin

**Supplementary Figure 1. A flowchart for patient enrollment and follow-up.**

IL, interleukin.


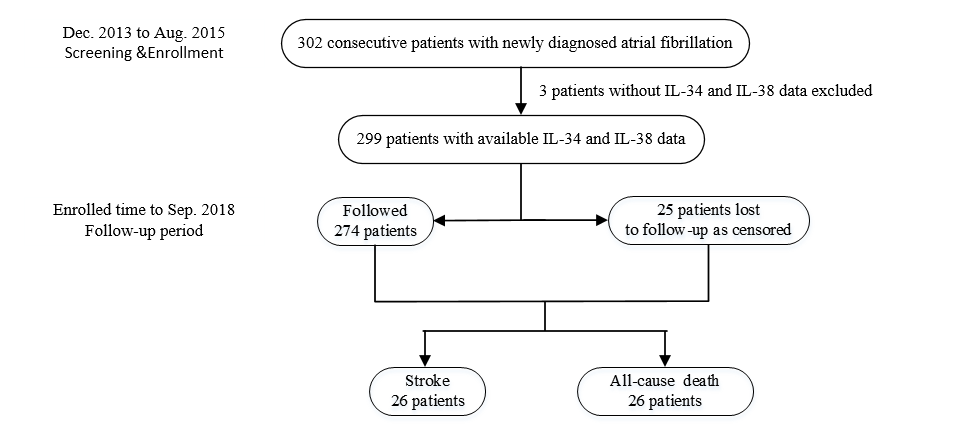

Supplement: Supplementary file 1 [file Data_Sheet_1.docx]
